# Supplementary material for: Tolerance interval testing for assessing accuracy and precision simultaneously
Source: PLoS One. 2021 Feb 5;16(2):e0246642. doi: 10.1371/journal.pone.0246642 (PMC7864420; doi:10.1371/journal.pone.0246642)
Supplement: S1 Appendix — (DOCX) [file pone.0246642.s001.docx]

## S1 Appendix. Derivation of the asymptotic normality of the lengths

It, it is well-known that, by Central Limit Theorem,

$\frac{S^{2}-\sigma^{2}}{\sigma^{2}\sqrt{2/\left(n-1\right)}}\to^{D} Z$.

And, via delta theorem, we have

$\frac{S-\sigma}{\sigma\sqrt{2\left(n-1\right)}}\to^{D} Z$.

However, since $\sqrt{n-1}S/\sigma$ follows a chi distribution, we know that the mean of $S$ is $\mu_{S}$, while the standard deviation is $\sigma_{S}$. To obtain a more accurate approximation, we use the Legendre duplication formula and Stirling’s approximation for gamma function and obtain

$\sqrt{2}\Gamma\left(n/2\right)/\Gamma\left(\left(n-1\right)/2\right)=\sqrt{n-1}\left[1-1/\left(2n\right)+O\left(n^{-2}\right)\right]$.

This leads to

$\frac{\sigma/\sqrt{2\left(n-1\right)}}{\sigma_{S}}\to1$ and $\sigma-\mu_{S}\to0$.

Consequently, by applying Slutsky theorem twice, we have

$\frac{S-\sigma}{\sigma/\sqrt{2\left(n-1\right)}}\frac{\sigma/\sqrt{2\left(n-1\right)}}{\sigma_{S}}+\left(\frac{\sigma-\mu_{S}}{\sigma_{S}}\right)=\frac{S-\mu_{S}}{\sigma_{S}}\to^{D} Z\times1-0=Z$.

Now, since $\bar{X}$ and $S$ are independent, the bivariate distribution is then

.

Since $L=\bar{X}-kS$ and $U=\bar{X}+kS$, we have

.

Consequently,

.
