# Supplementary material for: Tolerance interval testing for assessing accuracy and precision simultaneously
Source: PLoS One. 2021 Feb 5;16(2):e0246642. doi: 10.1371/journal.pone.0246642 (PMC7864420; doi:10.1371/journal.pone.0246642)
Supplement: S2 Appendix — (DOCX) [file pone.0246642.s002.docx]

## S2 Appendix. SAS code for sample size determination

/*--------------------------Parameters setting---------------------*/

/* Users should provide the following specifications of the parameters for sample size determination */

%let alpha=0.1; /*The significance level*/

%let gamma=0.9; /*The content level*/

%let power=0.8; /*The required power*/

%let tau=10; /*The acceptable limit*/

%let m=0; /*Mean*/

%let sd=3; /*Standard deviation*/

**%macro** ***para***;

sigma=&sd****2**;

k=sqrt(quantile("normal", (**1**+&gamma)/**2**)****2**/quantile("chisq", &alpha, n-**1**)*(n-**1**)*(**1**+**1**/n));

m_s=sqrt(sigma***2**/(n-**1**))*gamma(n/**2**)/gamma((n-**1**)/**2**);

v_s=sigma*(**1**-**2***(gamma(n/**2**)/gamma((n-**1**)/**2**))****2**/(n-**1**));

m1=&tau+&m-k*m_s;

m2=&tau-&m-k*m_s;

sigmaa=sigma/n+k****2***v_s;

rho=(k****2***v_s-sigma/n)/(k****2***v_s+sigma/n);

APower=probbnrm(m1/sqrt(sigmaa), m2/sqrt(sigmaa), rho);

**%mend**;

**proc** **nlp** outest=aaa noprint;

min nn;

decvar n=**10**;

bounds n>=**5**;

nlincon APower=&power;

%***para***

nn=n;

**run**;

**data** bbb; set aaa; if _type_="PARMS";

n=ceil(n);

%***para***

m=&m;

sd=&sd;

tau=&tau;

**run**;

**proc** **print** noobs;

var m sd tau n APower; **run**;
