# Supplementary material for: Tolerance interval testing for assessing accuracy and precision simultaneously
Source: PLoS One. 2021 Feb 5;16(2):e0246642. doi: 10.1371/journal.pone.0246642 (PMC7864420; doi:10.1371/journal.pone.0246642)
Supplement: S3 Appendix — (DOCX) [file pone.0246642.s003.docx]

## S3 Appendix. SAS code for p-value calculation

/*--------------------------Parameters setting---------------------*/

/* Users should provide the following specifications of the parameters for sample size determination */

%let alpha=0.1; /*The significance level*/

%let gamma=0.9; /*The content level*/

%let power=0.8; /*The required power*/

%let tauL=980; /*The lower acceptable limit*/

%let tauU=1020; /*The upper acceptable limit*/

%let m=1000; /*Mean*/

%let sigma=20; /*Standard deviation*/

%let n=9; /*Sample size*/

%let l=981.2; /*The observed lower tolerance bound*/

%let u=1004.5; /*The observed upper tolerance bound*/

**%macro** ***ev***;

%do c=**1** %to **3**;

proc nlp out=aaa&c;

max pvalue;

decvar m=&m, sigma=&sigma;

nlincon %if &c=**1** %then %do; h01=&tauL %end;

%if &c=**2** %then %do; h02=&tauU %end;

%if &c=**3** %then %do; h01=&tauL, h02=&tauU %end;;

k=sqrt(quantile("normal", (**1**+&gamma)/**2**)****2**/quantile("chisq", &alpha, &n-**1**)*(&n-**1**)*(**1**+**1**/&n));

m_s=sqrt(sigma***2**/(&n-**1**))*gamma(&n/**2**)/gamma((&n-**1**)/**2**);

v_s=sigma*(**1**-**2***(gamma(&n/**2**)/gamma((&n-**1**)/**2**))****2**/(&n-**1**));

m1=-&l+m-k*m_s;

m2=&u-m-k*m_s;

sigmaa=sigma/&n+k****2***v_s;

rho=(k****2***v_s-sigma/&n)/(k****2***v_s+sigma/&n);

pvalue=probbnrm(m1/sqrt(sigmaa), m2/sqrt(sigmaa), rho);

h01=m-quantile("normal", &gamma)*sqrt(sigma);

h02=m+quantile("normal", &gamma)*sqrt(sigma);

run;

%end;

**%mend**;

%***ev***

**data** aaa; set aaa1 aaa2 aaa3; sd=sqrt(sigma); **run**;

**proc** **print** data=aaa; format pvalue **7.4** h01 **7.4** h02 **7.4**;

var pvalue h01 h02 m sd; **run**;

**run**;
