# Supplementary material for: Tolerance interval testing for assessing accuracy and precision simultaneously
Source: PLoS One. 2021 Feb 5;16(2):e0246642. doi: 10.1371/journal.pone.0246642 (PMC7864420; doi:10.1371/journal.pone.0246642)
Supplement: Attachment — Submitted filename: PONE-D-20-31383_REVIEW.pdf [file pone.0246642.s004.pdf]

Comments on **A tolerance interval testing for assessing accuracy and precision simultaneously** (PONE-D-20-31383) by C. Chiang and M. Zhunan

Summary

This paper attempts to address a unifying inferential framework regarding tolerance intervals. Simulations and a real dataset are analyzed.

Overall Comments

Overall, I found this paper difficult to follow. I understand the basic premise of seeking a general unifying inferential framework for tolerance intervals, but the flow of the paper is uneven and details are often vague. I think more intuitive discussions about the sample size determination would be beneficial, and overall a more expansive power study and analysis of said results would strengthen the work. More detailed comments follow below.

Detailed Comments

1. Grammar tends to be an issue. Just three examples near the beginning of the paper include:

   - Page 1: "...small value of variation accommodated..." should be "...small value of variation, accommodating..."
   - Page 2: On the third line, "...variability of X is out of ours" does not make sense.
   - Page 5: On the first line, the part that states "...a two-sided..." should be the start of a new sentence.

   Thus, extensive revising is necessary.

2. More care needs to be taken with Equation (2). In particular, you need to show that the inner probability statement is a conditional statement, and you need to show which quantities the probability statements are being taken with respect to. See, for example, the definition in Krishnamoorthy and Mathew (2009).

3. While the USP guideline still use the Howe approximation, note that calculation of the exact k-factor is not prohibitive. For example, the `k.factor()` function in the `tolerance` package (Young, 2010) for `R` calculates the exact k-factor. This makes the discussion on Page 13 about using approximate calculations unnecessary.

4. Page 6: What is "the validation rule?"

5. Page 7: The transition in the power rule in Equation (5) to Equation (6) is not entirely clear. What is the equivalence in moving from the power rule in terms of c_L and c_U to writing it in terms of τ_L and τ_U?

6. Page 7: What are the df for the chi-square distribution of $\sqrt{n-1}S/\sigma$?

7. Page 7: The bivariate normal distribution of L and U needs more details about the derivations of the mean vector and covariance matrix. Since this is a critical point for how the rest of the inferential theory supposedly follows, the derivation needs to be explicitly clear.

8. Page 9: The first two sentences don't make sense. What is meant by "the maximum is used"?

References

Krishnamoorthy, K. and Mathew, T. (2009). *Statistical Tolerance Regions: Theory, Applications and Computations*. John Wiley.

Young, D. S. (2010). "tolerance: An R Package for Estimating Tolerance Intervals." *Journal of Statistical Software*, 36(5):1–39.
