# Supplementary material for: Tolerance interval testing for assessing accuracy and precision simultaneously
Source: PLoS One. 2021 Feb 5;16(2):e0246642. doi: 10.1371/journal.pone.0246642 (PMC7864420; doi:10.1371/journal.pone.0246642)
Supplement: Attachment — Submitted filename: Response to Reviewers.docx [file pone.0246642.s005.docx]

**Response to Comments on comments on Manuscript PONE-D-20-31383 “A tolerance interval testing for assessing accuracy and precision simultaneously” by Chieh Chiang and Chin-Fu Hsiao**

We sincerely thank the reviewers for their careful, constructive, thorough and thoughtful review which greatly improves the contents and presentation of our work. We have addressed all the comments in a point-to-point manner in the following and have incorporated all the referees’ suggestions into the revision.

**With respect to Reviewer #1’s comments,**

*The authors explain in this article how to calculate the power when using a 2-sided tolerance interval (to assess jointly the mean and the standard deviation (accuracy and precision)) and the corresponding statistical hypotheses are clarified.*

*This paper is well written and concise. The power function is built under asymptotical property (bivariate normal distribution) which is somewhat disappointing (especially that the tolerance intervals are very useful for small sample sizes), but the results from the simulations show a very good agreement between this approximate power and the empirical power. As a consequence, the theory described in this paper can be implemented (sas code is given by the authors) and will bring an added-value to the scientific community.*

**Response**: Thank you for the encouragement.

*Here are some remarks to improve the manuscript:*

*The authors should comment and explain what it is new in their paper compared to this one:*

*https://onlinelibrary.wiley.com/doi/epdf/10.1002/sim.8695*

**Response**: Chen and Hsiao’s paper is to assess the similarity between two populations. In contrast, this study focuses on the assessment for one population. So, we realize the study of Dong et al. is more appropriate to be mentioned in this paper; their study is also for one population. In fact, the ideas of two one-sided hypothesis testing of Chen and Hsiao’s paper and Dong et al. study are the same. However, Novick et al. had pointed out that the use of two one-sided tolerance intervals is correct for controlling of the type I error rate only if the variability of the population is sufficiently small. We have added “Tolerance interval approaches have been widely used in the area of sampling acceptance criteria, however, the relationship between hypothesis testing and tolerance interval sampling acceptance plan were scarcely discussed [3]. A hypothesis test is so important because, for example of drug development, the objects are often humans so that we must control type I error rate and achieve a sufficient power because of cost. Therefore, Novick et al. [2] and Dong et al. [3] suggested two one-sided tolerance interval tests to dose content uniformity tests, delivered dose uniformity tests, and dissolution tests. In their applications, the hypotheses $H_{0L}:\Pr(X<c_{L})\geq P_{1}$ and $H_{0U}:\Pr(X<c_{U})\geq P_{2}$ were tested, respectively, where $X$ is a random variable from a population with prespecified constants $c_{L}$, $c_{U}$, $P_{1}$, and $P_{2}$. However, Novick et al. had pointed out that the use of two one-sided tolerance intervals is correct for controlling of the type I error rate only if the variability of the population is sufficient small. If so, the use of the tolerance interval test seems to be meaninglessness since it is essentially equivalent to testing merely the population mean. Moreover, whether the variability of the population is small enough is usually unknown in practice.” in the introduction.

*In p17 (pdf) - p11 article*

*"First, for the 27 combinations of the parameters, all of the empirical powers are greater than the desired 80%, which demonstrates that the proposed sample size determination can provide sufficient power for validating both accuracy and precision simultaneously based on the two-sided tolerance interval." This is somewhat confusing, power should be calculated for a given set of parameters. Bayesian power should be calculated if one wants to take into account the uncertainty around the parameters. The authors should elaborate or re-explain this clearly.*

**Response**: Sorry for the confusion. We have changed the sentence to “First, for the 27 different sets of parameters, all of the empirical powers are greater than the desired 80%, which demonstrates that the proposed sample size determination can provide sufficient power under various sets of parameters for validating both accuracy and precision simultaneously based on the two-sided tolerance interval.”

*eq(6): define tau clearly*

**Response**: Sorry for the mistake. Equation (6) should be “$\pi(\mathbf{\theta})=P\left\{L>c_{L},U<c_{U}|\mathbf{\theta}\right\}$”.

*Dong et al. used a TOST approach with 1-sided intervals, but the 2-sided tolerance interval used in this paper is not new anyway. It is applied, for instance, in method comparison studies, and also in validation of analytical methods.*

*The approximate 2-sided tolerance interval by Howe has already been assessed in the statistical literature (many papers, and book).*

**Response**: We have added several descriptions in this paper to emphasis the importance of the connection between a tolerance interval and a hypothesis test. Use of tolerance intervals is not new, however, applying a tolerance interval to a hypothesis test is scarce. A hypothesis test is so important because, for example of drug development, the objects are often humans so that we must control type I error rate and achieve a sufficient power because of cost.

**With respect to Reviewer #2’s comments,**

*Reviewer #2: Overall, I found this paper difficult to follow. I understand the basic premise of seeking a general unifying inferential framework for tolerance intervals, but the flow of the paper is uneven and details are often vague. I think more intuitive discussions about the sample size determination would be beneficial, and overall a more expansive power study and analysis of said results would strengthen the work. More detailed comments follow below.*

**Response**: Sorry for letting you feel difficult to follow. We have rewritten the manuscript to improve the reading and the manuscript has been English edited by English editing company American Journal Experts. And, based on your suggestion, we have added “On the other hand, sample size determination for a tolerance interval is, traditionally, to achieve a desired width of the interval [6,7]. In doing so, only the control of precision is considered in traditional sample size determination of a tolerance interval. Now, if hypothesis testing and tolerance interval sampling acceptance plan is linked, to determine sample size for a desired power of a tolerance interval test is equivalent to provide sufficient large probability of the tolerance interval falling within a prespecified acceptance interval; that is, accuracy and precision are taken into consideration of sample size determination simultaneously. Therefore, to evaluate the required sample size for a two-sided tolerance interval test is also an important aim in this study.” in the introduction.

*Detailed Comments*

*1. Grammar tends to be an issue. Just three examples near the beginning of the paper include:*

*Page 1: “...small value of variation accommodated...” should be “...small value of variation, accommodating...”*

**Response**: We have corrected the sentence based on your suggestion.

*Page 2: On the third line, “...variability of X is out of ours” does not make sense.*

**Response**: Has modified to “...variability of X exceeds what expected”.

*Page 5: On the first line, the part that states “...a two-sided...” should be the start of a new sentence.*

*Thus, extensive revising is necessary.*

**Response**: We have changed the sentence based on your suggestion.

*2. More care needs to be taken with Equation (2). In particular, you need to show that the inner probability statement is a conditional statement, and you need to show which quantities the probability statements are being taken with respect to. See, for example, the definition in Krishnamoorthy and Mathew (2009).*

**Response**: We has modified the equation “$P\left[P\left(L<X<U\right)\geq\gamma\right]=1-\alpha$” to “$P_{L,U}\left[P_{X}\left(L<X<U|L,U\right)\geq\gamma\right]=1-\alpha$.”

*3. While the USP guideline still use the Howe approximation, note that calculation of the exact k-factor is not prohibitive. For example, the k.factor() function in the tolerance package (Young, 2010) for R calculates the exact k-factor. This makes the discussion on Page 13 about using approximate calculations unnecessary.*

**Response**: Thank you for the information of the R package. As we known, the k.factor() function, or other statistical software, has to assign a known sample size for the calculation of k. However, the sample size is often unknown at the design stage for a test and has to be evaluated by the proposed sample size determination formula. In this situation, we realized, an approximation with a closed-form can simplify the calculation of sample size. Hence, we have added the sentence “Currently, the calculation of the exact tolerance factor is not prohibitive. For example, the k.factor() function in the tolerance package [10] for R calculates the exact k-factor. However, it is known that the tolerance factor is a function of the sample size, while the required sample size is unknown for achieving a desired power and must be evaluated by the proposed sample size determination formula. Hence, an approximation of the tolerance factor with a closed-form can simplify the calculation.” in the discussion.

*4. Page 6: What is “the validation rule?”*

**Response**: We have changed “the validation rule” to “the rejection rule”.

*5. Page 7: The transition in the power rule in Equation (5) to Equation (6) is not entirely clear. What is the equivalence in moving from the power rule in terms of* $c_{L}$ *and* $c_{U}$ *to writing it in terms of* $\tau_{L}$ *and* $\tau_{U}$*?*

**Response**: Sorry for the mistake. Equation (6) should be “$\pi(\mathbf{\theta})=P\left\{L>c_{L},U<c_{U}|\mathbf{\theta}\right\}$”.

*6. Page 7: What are the df for the chi-square distribution of* $\sqrt{n-1}S/\sigma$*?*

**Response**: Sorry for the confusion. $\sqrt{n-1}S/\sigma$ should follow to a chi distribution with degrees of freedom $n-1$. We have added “... with degrees of freedom $n-1$” to the sentence.

*7. Page 7: The bivariate normal distribution of L and U needs more details about the derivations of the mean vector and covariance matrix. Since this is a critical point for how the rest of the inferential theory supposedly follows, the derivation needs to be explicitly clear.*

**Response**: We have added an appendix for the derivations.

*8. Page 9: The first two sentences don't make sense. What is meant by “the maximum is used?”*

**Response**: We have changed the sentences to “Note that, under the normal assumption, there are infinite combinations of means and standard deviations satisfying the null hypothesis (1). Hence, a Lagrange multiplier method is used to evaluate the maximum of *p*-value; we therefore provide an SAS code in Appendix 3.”
